# MiR-497-5p regulates ox-LDL-induced dysfunction in vascular endothelial cells by targeting VEGFA/p38/MAPK pathway in atherosclerosis

**DOI:** 10.1016/j.heliyon.2024.e28887

**Published:** 2024-03-27

**Authors:** Wei Lu, Guoqing Wan, He Zhu, Tao Zhu, Xinmei Zhang

**Affiliations:** a Department of Cardiovascular Surgery, The Quzhou Affiliated Hospital of Wenzhou Medical University, Quzhou People's Hospital, Quzhou, 324000, China; b School of Pharmacy, Shanghai University of Medicine and Health Sciences, Shanghai, China; c Zhejiang Chinese Medical University, Zhejiang, China

**Keywords:** Atherosclerosis, HUVECs, Ox-LDL, miR-497-5p, VEGFA, p38

## Abstract

**Background:**

The impairment of endothelial cells triggered by oxidized low-density lipoprotein (ox-LDL) stands as a critical event in the advancement of atherosclerosis (AS). MiR-497-5p has been recognized as a potential predictor for AS, but its precise involvement in ox-LDL-induced endothelial cell dysfunction remains to be elucidated.

**Methods:**

An *in vitro* AS cell model was established by exposing human umbilical vein endothelial cells (HUVECs) to 100 μg/mL ox-LDL for 24 h. The assessment of endothelial cell function included evaluating cell viability, caspase-3 activity, inflammatory factors, and oxidative markers. Molecular mechanisms were elucidated through quantitative real-time PCR, Western blot analysis, and luciferase reporter assays.

**Results:**

Our investigation revealed that exposure to ox-LDL led to an upregulation in miR-497-5p and p-p38 levels, while downregulating the expression of vascular endothelial growth factor A (VEGFA) and phosphorylated (p)-endothelial nitric oxide synthase (*p*-eNOS) in HUVECs. Ox-LDL exposure resulted in decreased cell viability and angiogenic capacity, coupled with increased apoptosis, inflammation, and oxidative stress in HUVECs, partially mediated by the upregulation of miR-497-5p. We confirmed VEGFA as a direct target of miR-497-5p. Interfering with VEGFA expression significantly reversed the effects mediated by miR-497-5p silencing in HUVECs exposed to ox-LDL.

**Conclusions:**

In summary, our findings demonstrate that miR-497-5p exacerbates ox-LDL-induced dysfunction in HUVECs through the activation of the p38/MAPK pathway, mediated by the targeting of VEGFA.

## 1 Introduction

Atherosclerosis (AS) is a chronic and intricate vascular disease that underpins a spectrum of cardiovascular and cerebrovascular conditions [1]. Its pathogenesis entails a multitude of factors, including hyperlipidemia, hypertension, and hyperglycemia, actively involving low-density lipoprotein (LDL) [2]. Various cell types, such as endothelial cells, smooth muscle cells, and monocytes, participate in AS development under adverse conditions like stress, inflammation, and hypoxia [3,4]. The endothelial injury theory, which views atheromatous plaque formation as a consequence of endothelial injury, is a prominent concept in AS pathogenesis [5]. Hence, a comprehensive understanding of endothelial cell dysfunction triggered by ox-LDL is imperative for the development of innovative therapeutic strategies against AS.

MicroRNAs (miRNAs) are small, conserved, noncoding RNA molecules approximately 20–22 nucleotides in length that play a vital role in post-transcriptional gene regulation by binding to the 3′-untranslated regions (3′-UTRs) of target genes, leading to mRNA degradation or translational repression [6]. MiRNA have been shown to be crucial regulators in various pathophysiological processes and cardiovascular diseases [7]. Previous research by Shan et al. [8] demonstrated the upregulation of miR-497-5p in the progression of AS in ApoE-deficient mice through microRNA microarray analysis. MiR-497-5p has also been identified as a predictor for major adverse cardiovascular events [9,10]. Functionally, miR-497-5p downregulation has been linked to the reduction of acute lung injury caused by sepsis, accomplished through the targeting of IL2RB [11]. Moreover, miR-497-5p-mediated suppression of C–C motif chemokine ligand 19 (CCL19) has been shown to alleviate high glucose-induced cell apoptosis, inflammation, and fibrosis in HK-2 cells [12]. Additionally, knockdown of miR-497-5p has been found to reverse apoptosis, inflammation, and oxidative damage in lipopolysaccharide (LPS)-treated BEAS-2B cells [13]. Despite these findings, the specific role and mechanism of miR-497-5p in the regulation of endothelial cell dysfunction during AS remain poorly understood.

Vascular endothelial growth factor A (VEGFA), a well-studied member of the VEGF family, plays a central role in endothelial cellular functions, both in physiological angiogenesis (formation of blood vessels during tissue re-vascularization) and pathological angiogenesis (associated with inflammation and microvascular occlusion) [14]. Previous studies have demonstrated that VEGFA promotes the migration of human dental pulp stem cells through the p38 MAPK signaling pathways [15]. Moreover, Fan et al. [16] provided direct evidence implicating p38 MAPK in mediating hypoxia-induced increase in VEGFA biosynthesis in human endothelial cells. Furthermore, vascular endothelial growth factor receptor-2 (VEGFR2) has been identified as a common target gene of miR-410-3p, miR-497-5p, and miR-2355-5p, which were upregulated in coronary artery disease (CAD)-endothelial colony-forming cells (ECFCs) [17]. In our previous work, bioinformatics analysis predicted VEGFA to be a downstream gene of miR-497-5p. However, the specific mechanistic role of VEGFA in the progression of AS necessitates further investigation.

Therefore, our present study focused on investigating the regulatory role of the miR-497-5p/VEGFA axis in ox-LDL-treated human umbilical vein endothelial cells (HUVECs), serving as an AS cell model. We hypothesized that downregulating miR-497-5p could attenuate ox-LDL-induced dysfunction through the VEGFA-mediated p38 MAPK signaling pathway. These findings may offer a potential novel therapeutic approach for the treatment of AS.

## 2 Materials and methods

### 2.1 Generation of AS cell model

Human umbilical vein endothelial cells (HUVECs) acquired from American Type Culture Collection (ATCC; Manassas, VA, USA) were cultivated in Dulbecco's modified Eagle's medium (DMEM) plus 10% FBS (Gibco, Carlsbad, CA, USA) and 1% antibiotics (Gibco) under 37 °C with 5% CO_2_. The ox-LDL group was generated by exposing HUVECs to 100 μg/mL ox-LDL (Solarbio) for 24 h [18,19] to study cellular responses.

### 2.2 Cell transfection

MiR-497-5p inhibitor (anti-miR-497-5p), miR-497-5p mimics (miR-497-5p), corresponding negative controls (anti-miR-NC, miR-NC), small interfering RNA against VEGFA (si-VEGFA), and siRNA negative control (si-NC) were synthesized by Genepharma (Shanghai, China). Oligonucleotide transfections were performed using Lipofectamine 2000 reagent (Invitrogen, ThermoFisher Scientific, Inc., Waltham, MA, USA) following standard protocols, with a 48-h incubation period.

### 2.3 Cell viability assay

At specified time points (0, 24 and 48 h), HUVECs were seed into 96-well plates (5 × 10^3^ cell/well) and treated wtih Cell Counting Kit-8 (CCK-8) reagents (10 μl/well) (Beyotime, Shanghai, China). Following a 2-h incubation at 37 °C, the absorbance was measured at 450 nm using a microplate reader (BioTek, Winooski, VT, USA).

### 2.4 ELISA assay

The culture supernatant from HUVECs was gathered and analyzed for the release of tumor necrosis factor α (TNF-α) and interleukin 1β (IL-1β) utilizing specific commercial Enzyme-linked immunosorbent assay (ELISA) Kits (R&D Systems, Minneapolis, MN, USA) following established protocols.

### 2.5 Caspase-3 activity assay

To assess cell apoptosis, caspase-3 activity was quantified employing a caspase-3 colorimetric assay kit (Abcam, Cambridge, UK) following the manufacturer's instructions. Optical density at 400 nm was measured using a microplate reader, and relative caspase-3 activity was normalized to the control group.

### 2.6 Analysis of cell oxidative stress

Cellular oxidative stress was assessed by quantifying superoxide dismutase (SOD) and malondialdehyde (MDA) production utilizing dedicated commercial kits sourced from Jiancheng Biotech (Nanjing, China), following the manufacturer's protocols.

### 2.7 Capillary-like tube formation assay

HUVECs (3 × 10^4^ cells/well) were plated onto 96-well cell culture plates precoated with Matrigel (BD Biosciences). After 48 h-incubation, images were captured using a digital camera attached to a Nikon phase-contrast microscope. Analysis was performed using ImageJ 64 open software (National Institutes of Health, Bethesda, USA) following standard protocols.

### 2.8 Dual-luciferase reporter assay

The VEGFA 3′-UTR sequences, both with wild-type and mutant miR-497-5p binding sites, were inserted into pmirGLO vectors (Promega, Shanghai, China) as per Target Scan predictions. These constructed vectors were then co-transfected into HUVECs along with miR-497-5p and a non-targeting control (miR-NC). After a 48-h incubation period, the relative luciferase activity was measured using the dual-luciferase reporter assay system (Promega).

### 2.9 Quantitative real-time PCR

Total RNA was extracted from HUVECs using TRIzol reagent (Invitrogen). For miR-497-5p detection, complementary DNA (cDNA) was synthesized using the Taqman miRNA Reverse Transcription Kit (Qiagen, Valencia, CA, USA) and then amplified with TransScript Green One-Step quantitative real-time PCR SuperMix (Qiagen). Subsequently, quantitative PCR (qPCR) analysis was performed using SYBR Green PCR Master Mix (Qiagen). To detect VEGFA mRNA, total RNA was reverse transcribed into cDNA using the High-Capacity cDNA Reverse Transcription Kit (Applied Biosystems, Foster City, CA, USA). Quantitative real-time PCR was conducted using SYBR Premix Ex (Qiagen). The specific primer sequences used were detailed in Table 1. The expression levels of miR-497-5p and VEGFA mRNA were normalized to U6 and GAPDH, respectively, using the 2^−ΔΔCt^ method [20].

**Table 1:** Primers for quantitative real-time PCR.

Gene	Forward (5’ - 3′)	Reverse (5’ - 3′)
MiR-497-5p	CCTTCAGCAGCACACTGTGG	CAGTGCAGGGTCCGAGGTAT
U6	CTCGCTTCGGCAGCACA	ACGCTTCACGAATTTGCGT
VEGFA	CTCACCAAGGCCAGCACATA	ATACCGGGATTTCTTGCGCT
GAPDH	GGTGAAGGTCGGAGTCAACG	GCATCGCCCCACTTGATTTT

### 2.10 Western blot assay

Total proteins from HUVECs were extracted using RIPA lysis buffer (Sigma-Aldrich, St. Louis, MO, USA) and quantified using the bicinchoninic acid assay (BCA) (Beyotime). Equivalent amounts of proteins (30 μg) were separated SDS-PAGE and transferred onto polyvinylidene difluoride (PVDF) membranes (Millipore, Billerica, MA, USA). After blocked with 5% skimmed milk for 2 h, the membranes were incubated overnight at 4 °C with primary antibodies against VEGFA, p-p38, p38, and GAPDH (Abcam, Cambridge, MA, USA). Subsequently, the membranes were incubated with horse radish peroxidase (HRP) conjugated secondary antibody (Abcam) for 2 h at room temperature. Immuno-reactive signals were detected using the enhanced chemiluminescent (ECL) chromogenic substrate (Beyotime).

### 2.11 Statistical analysis

All experiments were conducted in triplicate, and the data were analyzed using GraphPad software 8.0 (GraphPad Inc., San Diego, CA, USA). Results are presented as mean ± standard deviation (SD). Statistical comparisons between two groups were performed using Student's t-test, with a significance threshold set at *p* < 0.05 indicating a significant difference.

## 3 Results

### 3.1 Ox-LDL caused a decrease in miR-497-5p expression and an increase in VEGFA/p38/MAPK expression in HUVECs

In our initial assessment of ox-LDL-treated HUVECs, quantitative real-time PCR revealed a significant increase in miR-497-5p expression (Fig. 1A), whereas the expression of VEGFA notably decreased when compared to the control group (Fig. 1B). Furthermore, Western blot analysis verified that in HUVECs subjected to ox-LDL treatment, there was a decrease in VEGFA and phosphorylated eNOS levels, and an increase in phosphorylated p38 protein levels (Fig. 1C and D) in HUVECs following ox-LDL treatment. These results suggest a potential association between the increase in miR-497-5p and the decrease in VEGFA, indicating a possible link to ox-LDL-induced dysfunction in HUVECs.

**Fig. 1:**
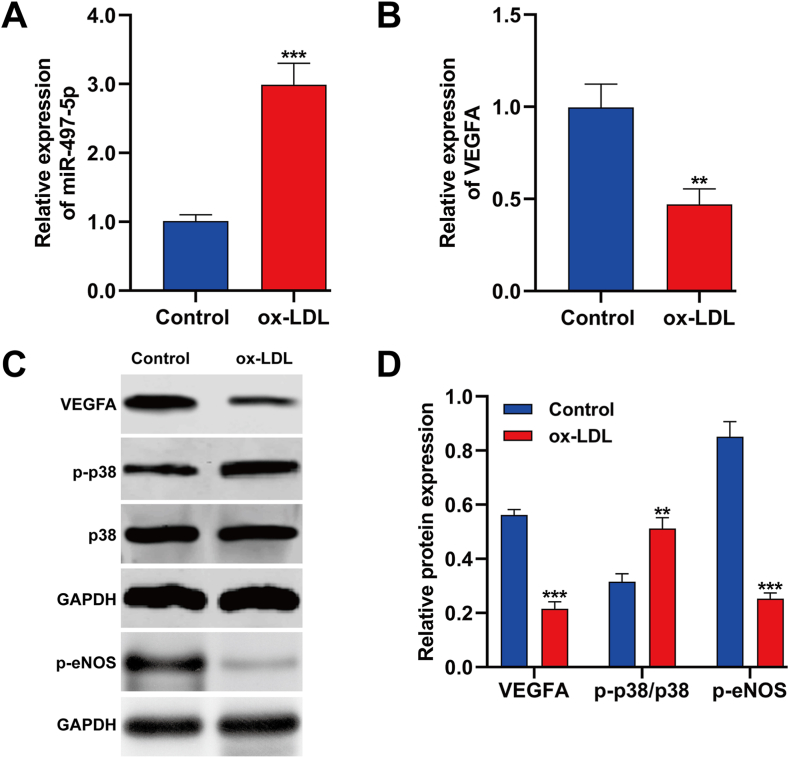
**Ox-LDL caused a decrease in miR-497-5p expression and an increase in VEGFA/p38/MAPK expression in HUVECs.** HUVECs were exposed to 100 μg/mL ox-LDL for 24 h. (A–B) Quantitative real time PCR analysis of miR-497-5p and VEGFA expression in ox-LDL induced HUVECs. (C–D) Western blot analysis of VEGFA, p-p38 and *p*-eNOS expression in ox-LDL induced HUVECs. All experiments were performed for three times with three technical repetitions. **p < 0.01, ***p < 0.001, compared with control.

### 3.2 Silencing of miR-497-5p largely alleviated ox-LDL-induced dysfunction in HUVECs

**Fig. 2:**
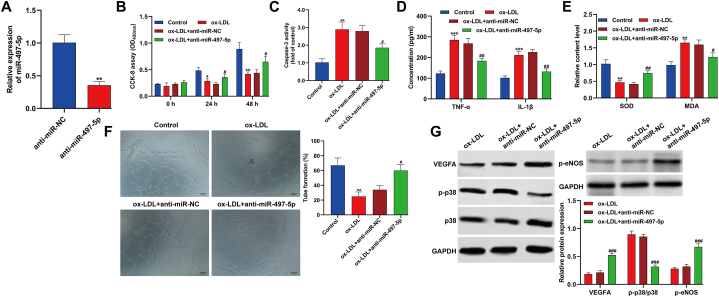
**Silencing of miR-497-5p largely alleviated ox-LDL-induced dysfunction in HUVECs.** HUVECs were transfected with anti-miR-497-5p or anti-miR-NC, followed by exposed to 100 μg/mL ox-LDL for 24 h. (A) Quantitative real time PCR was performed to evaluate the interference efficiency of anti-miR-497-5p in HUVECs. **p < 0.01, compared with anti-miR-NC; (B) CCK-8 assay was applied to analyze cell viability in different time points. (C) Caspase-3 activity was determined in HUVECs. (D) The concentrations of inflammation-associated cytokines (TNF-α and IL-1β) were detected by ELISA. (E) The production of SOD and MDA was assessed using their matching kits. (F) The ability of tube formation was analyzed through capillary-like network formation assay *in vitro*. (G) The protein levels of VEGFA, p-p38, p38 and *p*-eNOS were measured by Western blot assay. All experiments were performed for three times with three technical repetitions. *p < 0.05, **p < 0.01, ***p < 0.001, compared with control; #*p* < 0.05, ##*p* < 0.01. ###*p* < 0.001, compared with ox-LDL + anti-miR-NC.

Next, we utilized quantitative real-time PCR to confirm the effective silencing of miR-497-5p in HUVECs using anti-miR-497-5p (Fig. 2A). Our results demonstrated that knockdown of miR-497-5p significantly enhanced cell viability and reduced apoptosis in HUVECs exposed to ox-LDL, as depicted in Fig. 2B and C. Furthermore, the ox-LDL-induced inflammatory response (Fig. 2D) and oxidative stress (Fig. 2E) in HUVECs were notably mitigated upon miR-497-5p silencing. Additionally, the impaired ability of tube formation in ox-LDL-treated HUVECs was largely restored following miR-497-5p inhibition, as illustrated in Fig. 2F. Our findings also revealed that the reduction in VEGFA and *p*-eNOS expression, along with the activation of p-p38 by ox-LDL, were mitigated when HUVECs were treated with anti-miR-497-5p (Fig. 2G). Collectively, our data suggest that the inhibiting miR-497-5p significantly mitigated the dysfunction in HUVECs caused by ox-LDL.

### 3.3 MiR-497-5p overexpression exacerbated ox-LDL-induced impairment in HUVECs

**Fig. 3:**
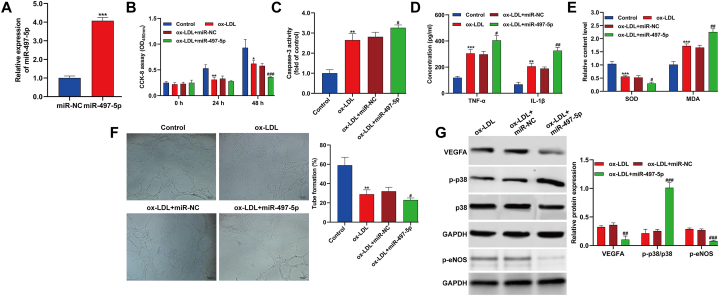
**MiR-497-5p overexpression exacerbated ox-LDL-induced impairment in HUVECs.** HUVECs were transfected with miR-497-5p mimics or miR-NC, followed by exposed to 100 μg/mL ox-LDL for 24 h. (A) Quantitative real time PCR was performed to evaluate the interference efficiency of miR-497-5p mimics in HUVECs. ***p < 0.001, compared with miR-NC; (B) CCK-8 assay was applied to analyze cell viability in different time points. (C) Caspase-3 activity was determined in HUVECs. (D) The concentrations of inflammation-associated cytokines (TNF-α and IL-1β) were detected by ELISA. (E) The production of SOD and MDA was assessed using their matching kits. (F) The ability of tube formation was analyzed through capillary-like network formation assay *in vitro*. (G) The protein levels of VEGFA, p-p38, p38 and *p*-eNOS were measured by Western blot assay. All experiments were performed for three times with three technical repetitions. **p < 0.01, ***p < 0.001, compared with control; #*p* < 0.05, ##*p* < 0.01, ###*p* < 0.001, compared with ox-LDL + miR-NC.

Transfecting HUVECs with miR-497-5p mimics significantly elevated miR-497-5p levels (Fig. 3A). Functional assays using CCK-8 and caspase-3 activity demonstrated that ox-LDL treatment led to reduced cell viability and increased apoptosis; these effects were exacerbated by miR-497-5p overexpression (Fig. 3B and C). Additionally, miR-497-5p overexpression intensified ox-LDL-induced inflammatory responses (Fig. 3D) and oxidative stress (Fig. 3E) in HUVECs. Ox-LDL exposure hindered cell angiogenic potential, and miR-497-5p overexpression further suppressed HUVEC tube formation ability (Fig. 3F). Western blot analysis revealed that overexpression of miR-497-5p intensified the reduction of VEGFA and and *p*-eNOS, and the increase of p-p38 levels in HUVECs treated with ox-LDL (Fig. 3G). In summary, these findings demonstrated that miR-497-5p overexpression intensified ox-LDL-induced dysfunction in HUVECs.

### 3.4 VEGFA was a downstream target of miR-497-5p in HUVECs

**Fig. 4:**
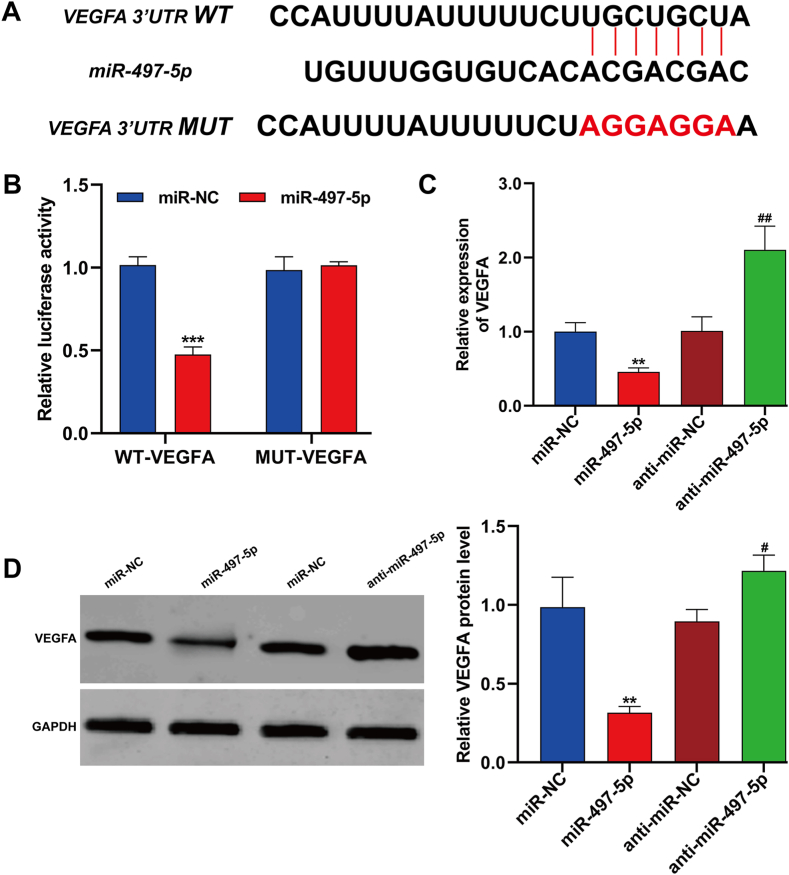
**VEGFA was a downstream target of miR-497-5p in HUVECs.** (A) The putative binding sites between miR-497-5p and VEGFA were predicted. (B) Dual-luciferase reporter assay in HUVECs after the co-transfection with WT-VEGFA or MUT-VEGFA and miR-497-5p or miR-NC or anti-miR-497-5p or anti-miR-NC was conducted. ***p < 0.001, compared with miR-NC; (C–D) The expression of VEGFA mRNA and protein was detected using quantitative real time PCR and Western blot analysis in HUVECs transfected with miR-497-5p or anti-miR-497-5p or the corresponding negative controls. All experiments were performed for three times with three technical repetitions. **p < 0.01, ***p < 0.001, compared with miR-NC; #*p* < 0.05, ##*p* < 0.01, compared with anti-miR-NC.

The Target Scan program analysis revealed a potential binding sequence between miR-497-5p and VEGFA, illustrated in Fig. 4A. Subsequent experimentation using a wild-type luciferase reporter plasmid (WT-VEGFA) demonstrated a significant reduction in luciferase activity upon miR-497-5p overexpression (Fig. 4B). Further investigation revealed a downregulation of both VEGFA mRNA and protein expression following miR-497-5p overexpression, as evidenced in Fig. 4C and D. Conversely, inhibiting miR-497-5p led to an increase in VEGFA expression. In summary, these findings provide compelling evidence that miR-497-5p directly interacts with VEGFA, exerting a negative regulatory effect on VEGFA expression in HUVECs.

### 3.5 Knockdown of VEGFA mitigated the impact of anti-miR-497-5p on ox-LDL-induced dysfunction in HUVECs

**Fig. 5:**
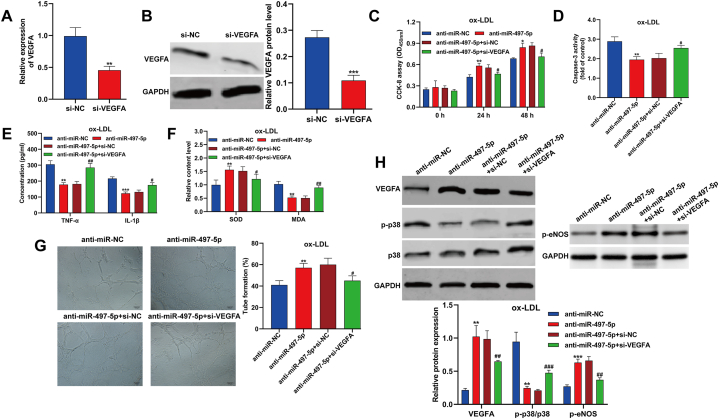
**Knockdown of VEGFA mitigated the impact of anti-miR-497-5p on ox-LDL-induced dysfunction in HUVECs.** (A–B) The interference efficiency of si-VEGFA in HUVECs was tested by quantitative real time PCR and Western blot analysis. **p < 0.01, ***p < 0.001, compared with si-NC; HUVECs were co-transfected with anti-miR-497-5p or anti-miR-NC and si-VEGFA or si-NC, followed by exposed to 100 μg/mL ox-LDL for 24 h. (C) CCK-8 assay was applied to analyze cell viability in different time points. (D) Caspase-3 activity was determined in HUVECs. (E) The concentrations of inflammation-associated cytokines (TNF-α and IL-1β) were detected by ELISA. (F) The production of SOD and MDA was assessed using their matching kits. (G) The ability of tube formation was analyzed through capillary-like network formation assay *in vitro*. (H) The protein levels of VEGFA, p-p38, p38 and *p*-eNOS were measured by Western blot assay. All experiments were performed for three times with three technical repetitions. *p < 0.05, **p < 0.01, ***p < 0.001, compared with anti-miR-NC; #*p* < 0.05, ##*p* < 0.01, ###*p* < 0.001, compared with anti-miR-497-5p + si-NC.

Transfecting HUVECs with si-VEGFA led to a notable reduction in VEGFA expression at both mRNA and protein levels, as depicted in Fig. 5A and B. This VEGFA silencing resulted in decreased cell viability (Fig. 5C) and induced apoptosis (Fig. 5D) in miR-497-5p-silenced HUVECs under ox-LDL exposure. Notably, the silencing of miR-497-5p protected HUVECs from ox-LDL-induced inflammation (Fig. 5E) and oxidative stress (Fig. 5F). However, these protective effects were reversed upon VEGFA silencing. Moreover, VEGFA silencing impaired the angiogenic ability of miR-497-5p-silenced HUVECs under ox-LDL exposure (Fig. 5G). Consistent with these findings, Western blot analysis revealed that VEGFA silencing counteracted the effects mediated by miR-497-5p knockdown on the expression of VEGFA, *p*-eNOS and p-p38 in ox-LDL-induced HUVECs (Fig. 5H). In summary, the downregulation of miR-497-5p protected HUVECs from ox-LDL-induced dysfunction, partially attributed to the upregulation of VEGFA.

## 4 Discussion

Our study reveals a new role for miR-497-5p in atherosclerosis (AS) development, showing its increase in HUVECs after ox-LDL exposure, linked with more inflammation, less *p*-eNOS, and more oxidative stress. *p*-eNOS is vital for heart health, affecting nitric oxide (NO) production, and oxidative stress from ox-LDL harms it, worsening heart disease risk [[21], [22], [23]]. MiR-497-5p helps regulate genes, and we found ox-LDL affects it mostly through oxidative stress, triggering pathways like NF-κB and MAPK that change miRNA levels, including miR-497-5p [24,25]. However, ox-LDL's effect on miR-497-5p might also involve lipid changes or other stress pathways [26,27], showing the complex ways ox-LDL and miR-497-5p interact in endothelial cells, with oxidative stress being a key but not the only factor.

Our experiments shed light on miR-497-5p′s impact on endothelial cells, particularly regarding the negative effects of ox-LDL in HUVECs. Suppressing miR-497-5p helped counteract the harmful impacts of ox-LDL, improving cell health and function, decreasing inflammation and oxidative stress, and boosting eNOS activation. Conversely, increasing miR-497-5p levels worsened ox-LDL's effects, highlighting how endothelial dysfunction, including abnormal cell growth and death, is central to AS [28]. In AS, high cholesterol triggers endothelial injury, leading to more lipids entering the bloodstream and forming plaques [29]. Ox-LDL, a cholesterol byproduct, is particularly damaging as it hinders endothelial cell growth by blocking vital growth factors [30]. Our results align with research showing that blocking miR-497-5p can reverse endothelial cell damage in different contexts [31], and with studies identifying miR-497-5p as a key microRNA in endothelial cells from patients with coronary artery disease [17]. These findings suggest miR-497-5p is involved in ox-LDL-related endothelial dysfunction, offering new insights into its role in AS development.

Our study discovered that miR-497-5p can bind to a specific part of the VEGFA gene, affecting how endothelial cells function, especially when they're damaged by ox-LDL, a harmful cholesterol byproduct. Prior research has highlighted VEGFA's critical role in endothelial cell damage and AS. For example, a reduction in VEGFA expression in ox-LDL-treated HUVECs and HASMCs hinted at VEGFA's potential protective role against cell injury induced by ox-LDL [32,33]. Our study corroborates this by showing that VEGFA knockdown negates the protective effects of anti-miR-497-5p on ox-LDL-induced HUVEC damage, suggesting VEGFA's contributory role in AS. Consistent with our results, miR-497-5p is considered a potential CAD biomarker due to its elevated levels in CAD patients' plasma, where it targets VEGFR2 [17]. Our data additionally establish VEGFA as a direct target of miR-497-5p. VEGF, a key angiogenesis regulator, primarily interacts with VEGFR2-mediated signaling in endothelial cells [34]. Generally, VEGF binding to VEGFRs triggers various intracellular signals, including MAPK activation in endothelial cells [35]. The p38 MAPK pathway has been linked to ox-LDL-induced endothelial cell apoptosis [36,37]. Our findings further suggest that miR-497-5p exacerbates ox-LDL-induced HUVEC damage and activates the p38 MAPK pathway. Comparable studies show miR-124 involvement in vessel endothelial cell apoptosis and proliferation inhibition via the p38/MAPK pathway [38]. Consequently, we hypothesize that the MAPK pathway contributes to the increased apoptosis, inflammation, and oxidative stress in ox-LDL-stimulated endothelial cells, influenced by miR-497-5p.

Our research strongly suggests that miR-497-5p is a key factor in the damage ox-LDL causes to HUVECs, especially through the VEGFA/p38/MAPK pathway. However, there are areas we didn't fully explore, such as examining more factors that cause inflammation, developing a real-life model for AS to confirm our findings, and looking into different pathways like NLRP3 inflammasome and PPARγ/CD36. Despite these gaps, our results indicate that focusing on miR-497-5p could offer a new approach to treating blood vessel problems in AS.

## Declarations

### 4.1 Ethics statement

This research did not involve any clinical samples or animal experiments, and therefore, the applicability of these considerations is not relevant.

## Consent for publication

Not applicable.

## Funding

This work is supported by the Ministry of Education Huatong Guokang Medical Research Project 2023 (NO. 2023HT092).

## Data availability

The data used to support the findings of this study are available from the corresponding author upon request.

## CRediT authorship contribution statement

**Wei Lu:** Software, Methodology, Formal analysis, Data curation. **Guoqing Wan:** Software, Methodology, Formal analysis, Data curation. **He Zhu:** Visualization, Validation, Software, Investigation. **Tao Zhu:** Writing – original draft, Validation, Methodology, Formal analysis. **Xinmei Zhang:** Writing – review & editing, Validation, Investigation, Funding acquisition, Conceptualization.

## Declaration of competing interest

The authors declare that they have no known competing financial interests or personal relationships that could have appeared to influence the work reported in this paper.

## Figures and Tables

**figs1:**
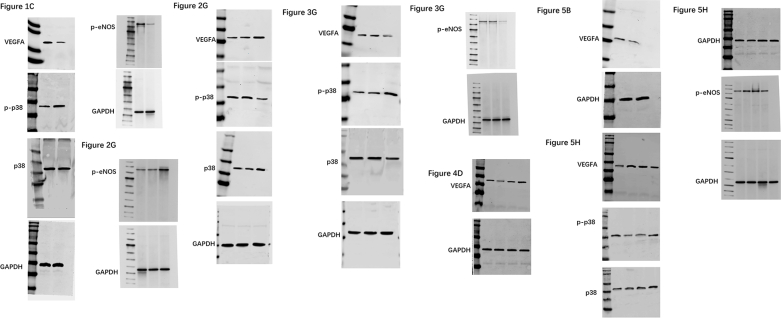


## References

[1] Libby P. (2021). The changing landscape of atherosclerosis. Nature.

[2] Di Pietro N., Formoso G., Pandolfi A. (2016). Physiology and pathophysiology of oxLDL uptake by vascular wall cells in atherosclerosis. Vascul Pharmacol.

[3] Crowther M.A. (2005). Pathogenesis of atherosclerosis. Hematology Am Soc Hematol Educ Program.

[4] Tabas,Garcia-Cardena,Guillermo,Owens,Gary, K. Recent Insights into the Cellular Biology of Atherosclerosis.. 10.1083/jcb.201412052 PMC4395483 25869663

[5] Mannarino E., Pirro M. (2008). Endothelial injury and repair: a novel theory for atherosclerosis. Angiology.

[6] Bartel D.P. (2004). MicroRNAs: genomics, biogenesis, mechanism, and function. Cell.

[7] Quiat D., Olson E.N. (2013). MicroRNAs in cardiovascular disease: from pathogenesis to prevention and treatment. J. Clin. Invest..

[8] Shan Z., Yao C., Li Z.L., Teng Y., Li W., Wang J.S., Ye C.S., Chang G.Q., Huang X.L., Li X.X. (2013). Differentially expressed microRNAs at different stages of atherosclerosis in ApoE-deficient mice. Chin Med J (Engl)..

[9] Osmak G., Baulina N., Kiselev I., Favorova O. (2021). MiRNA-Regulated pathways for Hypertrophic Cardiomyopathy: network-Based approach to insight into pathogenesis. Genes.

[10] Chen T., Zhang X., Qian W., Zhou R., Su M., Ma Y. (2022). Serum miR-497-5p serves as a diagnostic biomarker for acute coronary syndrome and predicts the occurrence of major adverse cardiovascular events after percutaneous coronary intervention. Bioengineered.

[11] Lou W., Yan J., Wang W. (2021). Downregulation of miR-497-5p Improves sepsis-induced acute lung injury by targeting IL2RB. BioMed Res. Int..

[12] Zhang D., Chen X., Zheng D. (2022). A novel MIR503HG/miR-497-5p/CCL19 Axis regulates high glucose-induced cell apoptosis, inflammation, and fibrosis in human HK-2 cells. Appl. Biochem. Biotechnol..

[13] Ke J., Chen M., Ma S., Zhang L., Zhang L. (2022). Circular RNA VMA21 ameliorates lung injury in septic rat via targeting microRNA-497-5p/CD2-associated protein axis. Bioengineered.

[14] Dabravolski S.A., Khotina V.A., Omelchenko A.V., Kalmykov V.A., Orekhov A.N. (2022). The role of the VEGF family in atherosclerosis development and its potential as treatment targets. Int. J. Mol. Sci..

[15] Sun X., Meng L., Qiao W., Yang R., Gao Q., Peng Y., Bian Z. (2019). Vascular endothelial growth factor A/Vascular endothelial growth factor receptor 2 axis promotes human dental pulp stem cell migration via the FAK/PI3K/Akt and p38 MAPK signalling pathways. Int. Endod. J..

[16] Fan B., Wang Y.X., Yao T., Zhu Y.C. (2005). p38 Mitogen-activated protein kinase mediates hypoxia-induced vascular endothelial growth factor release in human endothelial cells. Sheng Li Xue Bao.

[17] Su S.H., Wu C.H., Chiu Y.L., Chang S.J., Lo H.H., Liao K.H., Tsai C.F., Tsai T.N., Lin C.H., Cheng S.M. (2017). Dysregulation of vascular endothelial growth factor receptor-2 by Multiple miRNAs in endothelial colony-forming cells of coronary artery disease. J. Vasc. Res..

[18] Li C.Y., Ma L., Yu B. (2017). Circular RNA hsa_circ_0003575 regulates oxLDL induced vascular endothelial cells proliferation and angiogenesis. Biomed. Pharmacother..

[19] Qin X., Guo J. (2020). MicroRNA-328-3p Protects vascular endothelial cells against oxidized low-density lipoprotein induced injury via targeting Forkhead Box protein O4 (FOXO4) in atherosclerosis. Med Sci Monit.

[20] Livak K.J., Schmittgen T.D. (2001). Analysis of relative gene expression data using real-time quantitative PCR and the 2(-Delta Delta C(T)) Method. Methods.

[21] Chen C.A., Druhan L.J., Varadharaj S., Chen Y.R., Zweier J.L. (2008). Phosphorylation of endothelial nitric-oxide synthase regulates superoxide generation from the enzyme. J. Biol. Chem..

[22] Hung C.H., Chan S.H., Chu P.M., Lin H.C., Tsai K.L. (2016). Metformin regulates oxLDL-facilitated endothelial dysfunction by modulation of SIRT1 through repressing LOX-1-modulated oxidative signaling. Oncotarget.

[23] Ying W., Meiyan S., Wen C., Kaizu X., Meifang W., Liming L. (2023). Liraglutide ameliorates oxidized LDL-induced endothelial dysfunction by GLP-1R-dependent downregulation of LOX-1-mediated oxidative stress and inflammation. Redox Rep..

[24] Xu Z.R., Li J.Y., Dong X.W., Tan Z.J., Wu W.Z., Xie Q.M., Yang Y.M. (2015). Apple Polyphenols decrease atherosclerosis and Hepatic Steatosis in ApoE-/- mice through the ROS/MAPK/NF-kappaB pathway. Nutrients.

[25] Bao M.H., Li J.M., Zhou Q.L., Li G.Y., Zeng J., Zhao J., Zhang Y.W. (2016). Effects of miR-590 on oxLDL-induced endothelial cell apoptosis: Roles of p53 and NF-kappaB. Mol. Med. Rep..

[26] Liu Y., Jiang G., Lv C., Yang C. (2022). miR-222-5p promotes dysfunction of human vascular smooth muscle cells by targeting RB1. Environ. Toxicol..

[27] Wang Y., Chen X., Lu Z., Lai C. (2022). Circ_0093887 regulated ox-LDL induced human aortic endothelial cells viability, apoptosis, and inflammation through modulating miR-758-3p/BAMBI axis in atherosclerosis. Clin. Hemorheol. Microcirc..

[28] Gimbrone M.A., Topper J.N., Nagel T., Anderson K.R., Garcia-Cardena G. (2000). Endothelial dysfunction, hemodynamic forces, and atherogenesis. Ann. N. Y. Acad. Sci..

[29] Grover-Paez F., Zavalza-Gomez A.B. (2009). Endothelial dysfunction and cardiovascular risk factors. Diabetes Res. Clin. Pract..

[30] Chen C.H., Jiang W., Via D.P., Luo S., Li T.R., Lee Y.T., Henry P.D. (2000). Oxidized low-density lipoproteins inhibit endothelial cell proliferation by suppressing basic fibroblast growth factor expression. Circulation.

[31] Ma Q., Dai X., Lu W., Qu X., Liu N., Zhu C. (2021). Silencing long non-coding RNA MEG8 inhibits the proliferation and induces the ferroptosis of hemangioma endothelial cells by regulating miR-497-5p/NOTCH2 axis. Biochem. Biophys. Res. Commun..

[32] Zhang C., Wang L., Shen Y. (2021). Circ_0004104 knockdown alleviates oxidized low-density lipoprotein-induced dysfunction in vascular endothelial cells through targeting miR-328-3p/TRIM14 axis in atherosclerosis. BMC Cardiovasc. Disord..

[33] Cai T., Cui X., Zhang K., Zhang A., Liu B., Mu J.J. (2019). LncRNA TNK2-AS1 regulated ox-LDL-stimulated HASMC proliferation and migration via modulating VEGFA and FGF1 expression by sponging miR-150-5p. J. Cell Mol. Med..

[34] Law A.Y., Wong C.K. (2013). Stanniocalcin-1 and -2 promote angiogenic sprouting in HUVECs via VEGF/VEGFR2 and angiopoietin signaling pathways. Mol. Cell. Endocrinol..

[35] Hata Y., Miura M., Nakao S., Kawahara S., Kita T., Ishibashi T. (2008). Antiangiogenic properties of fasudil, a potent Rho-Kinase inhibitor. Jpn. J. Ophthalmol..

[36] Yin G., Yang X., Li B., Yang M., Ren M. (2014). Connexin43 siRNA promotes HUVEC proliferation and inhibits apoptosis induced by ox-LDL: an involvement of ERK signaling pathway. Mol. Cell. Biochem..

[37] Zhu Liao, Wang Ma (2001). Arteriosclerosis Thrombosis & Vascular Biology.

[38] Ma W., Zhang X., Liu Y. (2021). miR-124 promotes apoptosis and inhibits the proliferation of vessel endothelial cells through P38/MAPK and PI3K/AKT pathways, making it a potential mechanism of vessel endothelial injury in acute myocardial infarction. Exp. Ther. Med..

